# Preliminary Investigation of the Impact of Stress Suppression Processes and Counseling Strategies for Police Officers: A Qualitative Content Analysis

**DOI:** 10.3390/ijerph23020227

**Published:** 2026-02-10

**Authors:** Wen-Ling Hung

**Affiliations:** Department of Criminal Justice, Ming Chuan University, Taoyuan 333, Taiwan; wenling@mail.mcu.edu.tw

**Keywords:** police stress, emotional suppression, secondary trauma, aggressive behavior, psychological counseling strategy, qualitative content analysis

## Abstract

**Highlights:**

**Public health relevance—How does this work relate to a public health issue?**

- Police officers face chronic occupational stress that poses substantial risks to mental health, organizational functioning, and long-term well-being.
- Understanding stress-suppression behaviors provides insight into why officers may delay or avoid seeking timely psychological support.

**Public health significance—Why is this work of significance to public health?**

- This study identifies core psychological and institutional barriers that prevent police officers from accessing counseling services, a gap that directly affects workforce resilience and public safety.
- Findings contribute to the growing body of public health research focusing on high-risk first responders, emphasizing the need for early intervention and trauma-informed support systems.

**Public health implications—What are the key implications or messages for practitioners, policy makers and/or researchers in public health?**

- Results highlight the importance of developing evidence-based mental health programs tailored to policing culture, including confidential and stigma-free counseling pathways.
- Policymakers and practitioners may use these insights to strengthen preventive mental health strategies, reduce burnout, and improve organizational support mechanisms within law enforcement agencies.

**Abstract:**

(1) Background: With the increasing complexity of public safety duties, police officers are frequently exposed to high-pressure, high-risk environments. They face multiple stressors, including workload demands, societal expectations, supervisory pressure, and emergencies. Such factors can impair their mental health and emotional suppression capacity. (2) Methods: This preliminary qualitative investigation examines police officers’ perceptions of stress-related suppression processes through a literature review and semi-structured in-depth interviews with a small number of officers, employing qualitative content analysis. The research focuses on officers’ reported coping strategies, experiences with psychological counseling systems, and views on institutional mechanisms such as officer screening and emotional support structures. (3) Results: The findings reveal that participants reported generally lacking adequate emotional expression channels, leading to emotional dysregulation, outbursts, and burnout. Social support, supervisor attitudes, and flexible duty arrangements were perceived as key stress-mitigating resources. However, the utilization of current psychological counseling services remains low, primarily due to concerns regarding stigmatization and confidentiality. (4) Conclusions: This preliminary study recommends the development of a responsive mental health support framework for police agencies, emphasizing improvements in officer selection processes, mental health training, counseling accessibility, and organizational flexibility.

## 1. Introduction

Police work has long been recognized as a high-stress profession characterized by significant challenges, physical demands, mobility, temptations, and danger. Due to the wide range of service recipients, case complexity, and irregular working hours, police officers are frequently exposed to occupational violence and other potential risks. They often operate under conditions of elevated psychological stress, high exposure to violence, and a heightened risk of death [1]. Accordingly, policing is widely acknowledged in both practice and academia as a high-risk, high-stress occupation [2]. With pandemic prevention policies integrated into routine duties, police officers now bear additional responsibilities beyond maintaining public order and traffic control, such as pandemic containment and material distribution. These added duties have further increased workloads and psychological stress among police officers. Research indicates that stress-induced symptoms—such as insomnia, fatigue, and anxiety—are becoming increasingly prevalent [3]. Notably, frontline officers working under intense pressure may experience a deterioration of initially positive personality traits, e.g., enthusiasm for helping others and a strong sense of justice, which can manifest as emotional dysregulation and outbursts. Emotional dysregulation and outbursts hinder job performance and negatively impact emotional well-being and overall health [4].

Sources of stress in police work are multifaceted, including internal organizational pressure, challenges from the external social environment, inherent job risks, and personal psychological factors. When not effectively managed, stress can lead to a range of negative outcomes, including the deterioration of physical and mental health, personality changes, decreased work efficiency, and a disruption in family relationships [5]. This issue became particularly pronounced during the COVID-19 pandemic, during which rising infection rates significantly increased the psychological burden on frontline police officers. Studies show that compared to the general working population, frontline officers face higher psychological stress and are at greater risk for trauma, including posttraumatic stress disorder (PTSD) and secondary traumatic stress [6]. The SARS outbreak also revealed that numerous frontline workers in high-risk environments, such as nurses, experienced sleep disturbances and anxiety [7]. During COVID-19, officers tasked with quarantine enforcement and pandemic duties were frequently been subjected to public criticism, pressure from superiors, and emotional coercion from citizens, leading to pronounced emotional distress and psychological exhaustion [8].

Additionally, officers involved in major criminal cases and emergencies are more susceptible to emotional exhaustion and occupational burnout [9]. Prolonged exposure to stressors can result in depersonalization, reduced personal accomplishment, and emotional disturbances such as fear, grief, depression, and anxiety, often accompanied by guilt over failing to adequately protect the public [10]. When officers struggle with emotional regulation, it not only undermines their performance but also endangers their mental and physical health, weakens familial support systems, and threatens the stability of police operations [11]. Against this backdrop, there is a pressing need to better understand how police officers experience and cope with occupational stress, how stress influences their emotional regulation and inhibitory control in relation to aggressive behavior, and how existing counseling and organizational support mechanisms operate in practice. Therefore, the purpose of this study is to examine frontline officers’ stress experiences, emotional regulation and inhibitory control processes, and individual as well as organizational coping strategies, to explore their perceptions of current support systems, and to develop evidence-informed recommendations for screening, early intervention, and psychological support policies in police agencies.

## 2. Literature Review

Research suggests that frontline firefighters are more prone to sleep disorders and psychological distress, with a significant correlation observed between psychological stress and insomnia [12]. Similarly, emergency medical technicians (EMTs) and paramedics experience high rates of psychological distress, with studies indicating that depression and anxiety rates among EMS personnel are significantly higher than in other professions, and that approximately one-third meet diagnostic criteria for PTSD [13]. Given the similarities among the nature of police work, firefighting, and emergency medical services—all of which involve responding to emergencies and disasters, prolonged exposure to high stress, and unpredictable situations—police officers are similarly vulnerable. Without adequate mechanisms to release stress, frontline workers are likely to experience imbalances in physical and mental well-being, leading to problems across physiological, psychological, and behavioral domains.

### 2.1. Police Officers’ Responses to Stress

From a biological perspective, stress is defined as a reaction that occurs when an individual faces threats but lacks the immediate ability to respond [14]. Green et al. [15] found that early life stress (ELS) accounts for approximately 32% of mental health disorders and up to 44% of first-onset cases. Numerous studies also indicate that repeated exposure to ELS significantly increases the risk of mental health disorders and physical health issues [16,17,18,19,20,21,22,23]. Furthermore, ELS is associated with increased suicide risk. Dube et al. [24] reported that ELS accounts for up to 67% of the population attributable risk of suicide attempts. Anda et al. [25] and Brown et al. [26] found that adults who have experienced more than six ELS events may see their average lifespan reduced by approximately 20 years compared to those without such experiences.

Ivancevich and Matteson [27] categorized the concept of stress into three theoretical approaches:

#### 2.1.1. Stimulus-Based Approach

This perspective views stressors as external events. When individuals face changes, they must expend effort to adapt and restore equilibrium. The psychological tension generated during this process comprises the stress response.

#### 2.1.2. Response-Based Approach

According to Selye, stress arises from stimuli originating from various domains, including psychological, cultural, biological, and environmental sources. These stimuli trigger similar physiological responses, representing a nonspecific bodily adjustment process to stressors.

#### 2.1.3. Interaction-Based Approach

Lazarus and Folkman [28] emphasized that stress results from the interaction between individuals and their environment. Whether a stress response occurs depends on an individual’s cognitive appraisal of the stimulus as threatening and their perceived ability and resources to cope with it. If an individual perceives the threat but believes they can handle it, a stress response may not arise [29].

Based on the above, stress is not only influenced by external events but also regulated by an individual’s internal resources, cognitive assessment, and coping abilities. For police officers, prolonged exposure to a high-pressure working environment without adequate support and release mechanisms can significantly affect their physical and mental health as well as their performance on duty.

### 2.2. Secondary Traumatic Stress on Police Officers

When handling major criminal cases or disaster scenes, frontline police officers are routinely exposed to potentially traumatic events that may result in acute psychological distress. This can lead to what is known as secondary traumatic stress. This refers to the indirect psychological trauma experienced by professionals who work directly with trauma-exposed populations—including police officers, emergency responders, mental health clinicians, and social workers—through prolonged exposure to victims, their stories, or related traumatic incidents. These experiences—particularly involving critically injured individuals, death scenes, or graphic details—can readily trigger internalized trauma processes in police officers [30]. As frontline responders, police officers are continually exposed to violent, chaotic, and devastating environments, making them highly susceptible to secondary trauma responses, accumulated stress, and emotional exhaustion.

When such trauma occurs, negative emotions may intensify gradually. Without effective emotional regulation mechanisms, this can lead to deteriorating physical and psychological health [31]. For instance, research has identified significant correlations between traumatic stress and conditions such as insomnia and sleep disturbances [32]. Emotional exhaustion is closely linked to diminished organizational commitment and an increased risk of suicide. These indirect trauma effects are considered latent high-risk occupational factors, presenting symptoms similar to those of primary trauma, including anxiety, insomnia, emotional suppression, chronic fatigue, and exhaustion [33].

Using the Job Demands–Resources Model, Pappa et al. [34] demonstrated that when job demands exceed an individual’s available resources, the risk of burnout and emotional exhaustion increases significantly. Kim [35] emphasized that excessive job demands can exacerbate the psychological exhaustion associated with secondary trauma. Although the COVID-19 pandemic has remained relatively stable in Taiwan [36], the overwhelming media coverage and prolonged exposure of police officers to high-risk pandemic-related duties and public scrutiny have led to heightened infection anxiety and internalized stress [37], resulting in issues such as insomnia and emotional burnout [38].

The connection between stress and physical illness has been a long-standing research focus. For instance, Wollf [39] investigated the relationship between stress and headaches; Engel [40] examined stress and ulcers, while LeShan [41] explored its correlation with cancer; Friedman and Rosenman [42] investigated its association with heart disease. These studies have consistently shown that when occupational stress exceeds an individual’s coping capacity and is left unresolved, it can trigger substantial physiological and psychological responses. Lazarus and Folkman [43] highlight that individuals who lack effective coping strategies in the face of stressors are more likely to resort to emotion-focused coping. While this type of coping may offer short-term emotional relief, it can exacerbate problems over time, leading to more severe psychological distress and psychosomatic disorders.

Research indicates that negative coping mechanisms such as denial, self-blame, and alcohol abuse may provide short-term stress relief while ultimately worsening stress symptoms and can develop into serious mental health issues or social dysfunction [43]. Therefore, establishing robust psychological support systems and coping training mechanisms for frontline police officers is essential to enhance their emotional resilience and occupational well-being.

### 2.3. Aggressive Behavior-Related Neural Mechanisms

Aggressive behavior is typically classified into two types based on motivation and response patterns: proactive and reactive aggression [44,45]. In this study, these forms of aggression are viewed as behavioral manifestations of broader emotional dysregulation processes rather than as independent constructs.

#### 2.3.1. Proactive Aggressive Behavior

Proactive aggression is characterized by premeditated, goal-oriented, deliberate actions, often directed toward specific targets to obtain anticipated rewards, and can be regarded as a relatively less emotionally driven expression of dysregulated behavior. An example of this is predatory aggression. Such behavior is closely associated with activation of the prefrontal cortex (PFC) and is frequently accompanied by low levels of empathy and blunted sympathetic nervous system responses [46,47].

#### 2.3.2. Reactive Aggressive Behavior

Reactive aggression arises from emotionally charged stimuli such as provocation, threats, or loss, and represents a prominent affective expression of emotional dysregulation. When an individual anticipates a reward but fails to receive it, frustration may ensue. The OFC then heightens the activity of the amygdala and hypothalamus, triggering neural responses linked to aggression. In contrast, when outcomes match expectations, the system’s activity diminishes, inhibiting aggressive reactions [46,48]. Studies also suggest that antisocial personality traits are significantly associated with proactive aggression, while psychopathic tendencies may be related to both proactive and reactive aggression [48].

#### 2.3.3. Neurological Distinctions Between Aggression Types

Proactive aggression primarily relies on the regulatory functions of the prefrontal cortex; reactive aggression is associated with arousal in the limbic and hypothalamic systems, indicating a decline in prefrontal inhibitory control [46,47]. From the perspective of emotional dysregulation, this pattern reflects impaired top–down regulation of intense affective states. Reactive aggression is typically accompanied by intense sympathetic nervous system activation and emotional arousal, such as anxiety and anger [49].

#### 2.3.4. Psychological and Neurological Features of Reactive Aggression

Reactive aggression is often linked to anger, difficulty with emotional regulation, and provocative stimuli. Studies have found a significant correlation between reactive aggression and high levels of expressed anger [50,51,52]. These findings indicate that reactive aggression can be understood as a clinically salient manifestation of broader emotional dysregulation processes. Moreover, neuropsychological impairments are associated with tendencies toward reactive aggression, including emotional dysregulation, poor impulse control, threat hypersensitivity, cognitive empathy deficits, and reduced self–other differentiation. These characteristics are considered potential mechanisms driving reactive aggression and reflect specific psychological and neural facets of emotional dysregulation [53].

## 3. Research Design and Methods

This study conducted semi-structured interviews with seven police officers from diverse backgrounds, encompassing a range of years of service, genders, and ranks among frontline personnel (see Table 1 for participant characteristics). The research design aimed to capture a breadth of practical experiences and perspectives, analyzing stress coping mechanisms within and beyond the institutional context. The objective was to investigate the psychological and physiological responses to stress, emotional regulation, and the need for organizational support, enriching the depth and scope of the study.

### 3.1. Semi-Structured Interview Design and Procedure

This study conducted semi-structured interviews with seven police officers from diverse backgrounds, encompassing a range of years of service, genders, and ranks among frontline personnel. The research design aimed to capture many practical experiences and perspectives, analyzing stress coping mechanisms within and beyond the institutional context. The objective was to investigate the psychological and physiological responses to stress, emotional regulation, and need for organizational support, enriching the depth and scope of the study. A purposive sampling strategy was adopted to recruit frontline officers who had substantial experience with stress-inducing duties (e.g., patrol, criminal investigation, pandemic-related enforcement, or emergency response) and who were willing to participate in in-depth interviews about their stress, emotional regulation, and help-seeking experiences. Within these inclusion criteria, the researcher deliberately sought variation in rank, years of service, gender, and assignment type in order to capture a range of perspectives across different positions in the organizational hierarchy. However, the sample was confined to specific counties/cities and therefore does not cover all units within the Ministry of the Interior system.

#### 3.1.1. Conceptual Framework

**Figure 1:**
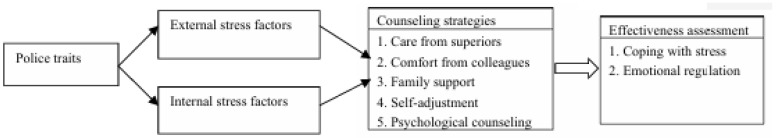
Conceptual study framework.

Based on the psychological counseling strategies currently adopted by police agencies, this study comprehensively examined factors that contribute to police stress, key police traits that shape stress responses (e.g., personality characteristics, emotional regulation capacity, and prior traumatic exposure), and the existing psychological counseling mechanisms. We conducted a focus group discussion to examine both individual and organizational coping strategies for such stress. The goal was to provide recommendations and strategies for enhancing the relevant counseling systems. The conceptual framework of this study, which links police traits, external and internal stressors, counseling strategies, and outcomes in terms of stress coping and emotional regulation, is illustrated in Figure 1.

1. Sources of stress: Psychological reactions and secondary traumatic stress

These include excessive workloads, sudden incidents, and pandemic-related duties; this elevates psychological stress levels and leads to insomnia, anxiety, and emotional exhaustion. Exposure to traumatic scenes or events may also trigger secondary traumatic stress.

2. Physical and mental health risks

Stress and secondary trauma increase the risk of physical and psychological conditions, such as depression, sleep disorders, and psychosomatic syndromes.

3. Aggressive behavior and neural mechanisms

Accumulated pressure and trauma may manifest as reactive or proactive aggression, each involving different neural structures (e.g., prefrontal cortex, amygdala, hypothalamus).

#### 3.1.2. Data Analysis Method: Thematic Analysis

This study used thematic analysis as the primary method for qualitative data analysis, following the six-phase framework proposed by Braun and Clarke [54]. The steps are as follows:

1. Familiarization with the data: The research team repeatedly read the interview transcripts to understand the context and meanings conveyed by the participants.
2. Generating initial codes: Open coding was conducted based on the interview content, marking recurring phrases and key semantic elements.
3. Searching for initial themes: Similar codes were grouped into thematic categories to develop an initial thematic structure.
4. Reviewing themes: The relationship between themes and the original data was cross-checked to ensure consistency; duplicate themes were merged or refined.
5. Defining and naming themes: Core concepts and boundaries of each theme were clarified and appropriately named to reflect their essence.
6. Producing the report: The results were synthesized; representative quotes are included for interpretive analysis and discussion.

To enhance analytic rigor, the researcher and a research assistant independently coded an initial subset of transcripts and then compared their coding to discuss discrepancies and refine the codebook. After consensus was reached, the refined codebook was applied to the remaining transcripts, and emergent codes were iteratively incorporated through team discussions. During theme review, the researcher repeatedly moved back and forth between coded extracts and full transcripts to confirm that each theme was strongly grounded in multiple participants’ accounts rather than in single, isolated quotations. Negative cases and deviant views were also examined to avoid over-simplified or overly homogeneous interpretations. Taken together, these procedures were implemented to enhance the transparency, credibility, and analytic objectivity of the qualitative findings despite the small sample size.

#### 3.1.3. Research Tools

The focus group interviews were used to comprehensively explore frontline officers’ experiences and coping mechanisms regarding cognitive conflict resolution, emotional regulation, and aggression management under stress. This study developed a semi-structured interview guide based on the research objectives and the following questions:

1. What are the common practical difficulties and challenges police officers face when handling cases?
2. What are the primary sources of stress experienced during emergency responses or routine duties?
3. What behavioral responses commonly occur when emotional regulation fails?
4. What mechanisms or support strategies does the organization adopt to handle or prevent violence-related incidents involving officers?
5. How do officers typically relieve stress in their daily duties? What are common coping methods?
6. What are the attitudes and actual practices of supervisors and organizational culture regarding “emotional regulation and stress coping among police officers”?
7. What are the specific observations and recommendations regarding inter-agency coordination, personnel training, staffing, and legal frameworks related to stress management and emotional regulation for police officers?

The interview guide balanced openness and guidance, fostering diverse experience-sharing and in-depth discussions while serving as the foundation for subsequent data analysis.

#### 3.1.4. Strategies for Ensuring Trustworthiness

We adopted the following strategies to ensure the reliability and validity of the collected data and analyses:

1. Triangulation: Cross-verification using data from participants with diverse service years, genders, and ranks, as well as through collaboration between the researcher and a research assistant, enhances data richness and consistency.
2. Peer review: Regular discussions between the researcher and a research assistant were conducted to verify the logic of coding and theme development.
3. Reflexivity: The researcher maintained reflective journals to assess the potential impact of personal roles, values, and interpretive positions on data comprehension. These reflections were regularly discussed with the research assistant and explicitly considered when making analytic decisions, in order to minimize the influence of preconceptions on data interpretation.
4. Member checking: Portions of the interview transcripts were reviewed and confirmed by participants to ensure that the narratives accurately reflected their original intentions, thereby enhancing data accuracy and representativeness. In addition, preliminary thematic summaries were shared with several participants, who provided feedback on whether the interpretations resonated with their experiences, leading to minor adjustments to theme boundaries and wording. These procedures were intended not only to enhance the overall credibility of the analysis, but also to make the influence of participants’ subjective perspectives more transparent and comparable across different cases.

#### 3.1.5. Research Limitations

This study is exploratory in nature and employed a small-scale qualitative interview design. While it offers in-depth insights into the subjective experiences of police officers under stress, its limitations are as follows:

1. Limited sample size: Only seven participants were interviewed, which restricts the generalizability of the findings and makes it challenging to represent the full spectrum of experiences among police personnel.
2. Geographically concentrated sample: Participants were recruited from police departments in specific counties/cities. Regional factors may have influenced the participants’ experiences, limiting the ability to account for nationwide institutional variations.
3. Risk of subjective interpretation: Qualitative analysis involves the researcher’s interpretation. Although triangulation and peer review were employed to enhance analytical validity, interpretations may still be influenced by the researcher’s positionality and cognitive biases. At the same time, officers with diverse ranks, years of service, and gender backgrounds were intentionally recruited to capture a range of experiences. The combination of triangulation, peer review, reflexive practice, and member checking was adopted to partially offset these limitations and to strengthen the robustness of the thematic patterns identified. In addition, officers’ personal experiences, emotional states at the time of the interview, and individual personality traits may have influenced how they chose to describe their stress and coping, and such subjective influences cannot be fully eliminated in a qualitative design, despite the use of triangulation, peer review, reflexivity, and member checking.
4. Lack of longitudinal data: The study was based on a single round of interviews and did not track the dynamic changes in stress responses or coping mechanisms over time. To address these limitations, and in line with the reviewer’s suggestion to expand the sample in future studies, subsequent research will aim to include a larger and more diverse police sample and to broaden the geographic coverage to different regions and units, including specialized divisions such as criminal investigation, traffic, and riot control. Future studies may also adopt mixed-methods or longitudinal designs that combine qualitative interviews with standardized psychometric measures or repeated surveys, in order to further validate and generalize the present findings and to examine the dynamic relationships among stress exposure, emotional regulation, and aggressive behavior over time.
5. A further limitation concerns the study’s temporal context. Data were collected during the COVID-19 pandemic, a period characterized by unprecedented operational challenges and heightened public health responsibilities that may have distinctively influenced officers’ experiences of occupational stress and emotional regulation. As such, the extent to which the findings generalize to non-pandemic policing environments warrants cautious interpretation and further empirical examination.

## 4. Research Findings and Discussion

### 4.1. Research Results and Analysis

**Figure 2:**
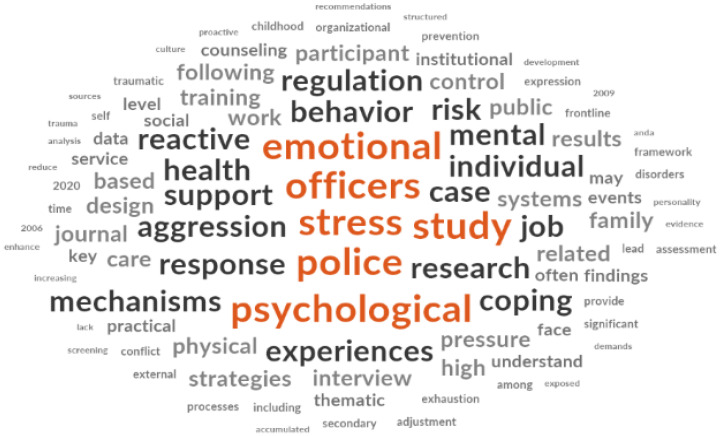
Word frequency chart of interview data.

Prior to presenting the detailed thematic findings, Figure 2 provides a word frequency overview of the interview data, generated using NVivo 14 software. The visualization highlights the prominence of terms such as “stress,” “pressure,” “work,” “emotion,” and “support” in participants’ accounts, offering an initial indication of the key conceptual domains addressed in the interviews. The following sections present the in-depth thematic analysis organized around these central concerns.

#### Facing Stress Challenges

1. Stress faced on duty

Due to the unique nature of their work, police officers are often under intense psychological stress. Routine tasks such as handling public reports, filling out paperwork, following orders from superiors, and engaging in crime prevention can foster considerable performance stress. Moreover, the stressors that frontline police officers face can vary depending on their personal aspirations for their role as police officers. As shown in Table 2, the thematic categories of stress faced on duty were derived from the interview data.

Summary:

The interview data reveal two primary dimensions of stress faced by frontline police officers:

- Discrepancy between public expectations and reality
  - The public holds high moral expectations for police officers, often conflicting with the limits of human capacity.
  - Media narratives tend to emphasize negative incidents, intensifying public criticism.

- Public interaction pressure
  - Officers often encounter doubt and confrontation during law enforcement.
  - Social media amplifies controversy, exacerbating psychological stress.
  - Police-citizen conflicts can erode public trust and morale.

**Figure 3:**
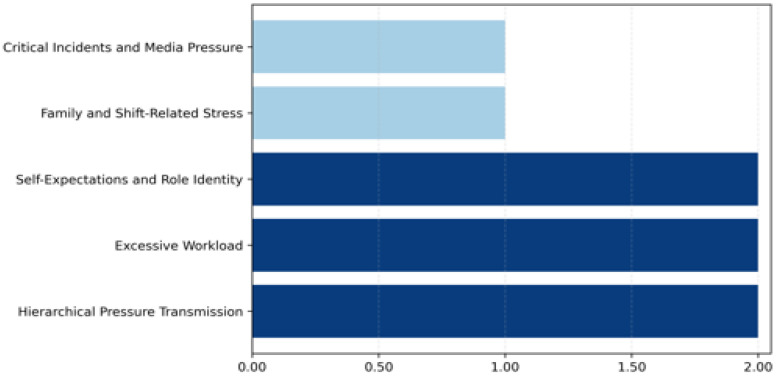
Stress hotspots among police officers interviewed.

These findings underscore the multifaceted nature of occupational stress in policing, encompassing both societal expectations and direct public interactions. As shown in Figure 3, hierarchical pressure transmission, excessive workload, and self-expectations and role identity emerged as the most prominent stressors, while family- and shift-related stress and critical incidents and media pressure were comparatively less frequently emphasized.

2. Role of CCTV in Police Investigations and the Associated Pressures on Police Officers

The effectiveness of closed-circuit television (CCTV) systems in crime prevention has long been a matter of scholarly contention. Nevertheless, within the realm of policing practice, CCTV plays an indispensable role in criminal investigations. Insights from this study’s focus group discussions reveal officers’ substantial dependence on CCTV footage during various stages of case investigation, particularly for evidence collection, suspect identification, and event reconstruction. However, investigative reconstruction becomes increasingly challenging when system coverage is insufficient or footage is improperly stored—especially in the absence of witnesses or physical evidence.

A salient finding emerging from the interviews concerns the lack of timely reporting by victims or other involved parties. Delayed notification frequently results in the automatic overwriting of crucial footage, thereby disrupting evidentiary continuity. Such delays substantially heighten investigative complexity and amplify the pressure placed on officers to reconstruct incident timelines and extract critical leads under conditions of evidentiary deficiency. Collectively, these CCTV-related constraints contribute to increased workloads, performance strain, and heightened psychological stress, underscoring the need for more systematic stress management and organizational support mechanisms within the policing context.As shown in Table 3, the thematic categories summarize the role of CCTV in police investigations and the associated pressures on police officers.

Summary:

Over-reliance on surveillance footage fosters structural limitations in modern policing. Statistics indicate that in over 85% of criminal investigations, CCTV is regarded as the primary source of initial evidence.

However, this dependency has led to overlooking traditional investigative methods such as trace evidence collection, interview techniques, and intelligence networking. When surveillance coverage is lacking or image quality is poor, investigators often struggle to proceed effectively.

3. Stress management patterns

- Stress control and emotional dysregulation behaviors

When police officers are unable to cope effectively with work-related stress or lack appropriate channels to process it, stress often manifests through their behaviors as forms of emotional expression. Past quantitative studies have suggested that individual characteristics—such as locus of control, psychological traits, and emotional quotient (EQ)—shape how stress is perceived, how well officers adapt to their work, and the extent of social support they receive, with more negative traits amplifying perceived stressors and increasing the risk of maladaptive responses. Consistent with these insights, the interview data in this study indicate that officers may experience physical, psychological, or behavioral difficulties in adaptation, which are reflected in various patterns of stress internalization and externalization. As shown in Table 4, the thematic categories summarize patterns of stress control and emotional dysregulation behaviors identified in the interview data.

4. Coping strategies

On the basis of the interview data, three main categories of coping strategies emerged, reflecting how officers respond to and manage work-related stress in their everyday practice. These categories encompass social support, professional support, and individual coping, and capture both formal and informal ways in which officers attempt to regulate emotions, maintain functioning, and adapt to organizational pressures. The following table summarizes these thematic categories, the associated subthemes, and illustrative insights from the interviews. As shown in Table 5, the thematic categories summarize the coping strategies police officers employ to manage work-related stress.

## 5. Conclusions and Suggestions

### 5.1. Research Conclusions

In interpreting the findings, the analysis distinguished between incident-based acute stress, chronic organizational and workload-related stress, and secondary traumatic stress arising from repeated exposure to traumatic scenes and victims’ suffering, using these categories as analytical lenses to better understand officers’ perceived emotional regulation and aggressive responses.

The first significant finding of this study indicates that stress and emotional regulation may serve as crucial foundations for suppressing impulsive and aggressive behavior. This finding is consistent with research demonstrating that emotion regulation difficulties are significantly associated with aggressive behavior [55], and that deficits in inhibitory control are closely linked to emotion-driven impulsivity [56].

This insight holds potential for application in pre-service police education and early intervention training. Prior research has shown that training programs targeting inhibitory control and emotion regulation skills can effectively reduce impulsive behaviors and enhance self-regulation capacity [57]. For instance, training programs targeting inhibitory functions and emotion regulation skills may help reduce the risk of impulsive actions during duty and improve officers’ ability to manage emotionally charged encounters with the public [38].

The second key finding addresses the challenges police officers face in stress coping and emotional regulation. The study provides recommendations at the organizational level. Interviewees generally agreed that personal psychological traits and emotional regulation abilities should be considered during recruitment. Once employed, institutions should offer more flexible leisure options and stress-relief mechanisms to reduce the accumulation of negative emotions instigated by workplace stress.

Based on the findings, this study proposes the following practical applications:

1. Screening mechanism for new recruits: We recommend the incorporating the assessments of emotional regulation abilities into the recruitment process to identify individuals prone to emotional dysregulation early while offering adequate psychological counseling and training resources.
2. Intervention for officers exhibiting aggressive behavior: For officers who have exhibited violent or emotionally dysregulated behavior during duty, we recommend psychological and neurological evaluations (including neurologic imaging and other studies if indicated) along with follow-up counseling and behavioral adjustment training. These intervention strategies are derived from the existing literature and the present qualitative findings, and their effectiveness in real-world settings will need to be empirically evaluated in future quantitative or mixed-methods studies.”
3. Monitoring and preventive measures for general officers: To understand the mental and emotional states of general officers, institutions should periodically conduct stress sampling and psychological stability assessments. This allows for timely support measures to strengthen resilience and workplace adaptability.

### 5.2. Research Suggestions

Based on the findings, this study proposes two primary areas of practical application: “Screening and Prevention Mechanisms” and “Stress Reduction and Support Strategies.” These suggestions are intended to guide police agencies in the planning of recruitment systems, training programs, and psychological support policies.

#### 5.2.1. Enhancing Officer Selection and Impulse Control Prevention Mechanisms

Constructing a “Self-Control Capacity Screening Framework” is recommended to better understand officers’ ability to manage emotional conflict and behavioral reactions in situations involving provocation and pressure. This framework should incorporate emotional regulation training and appropriate psychological and behavioral assessment tool to prevent emotional dysregulation and aggression during high-stress duties. Future studies can further refine and validate this framework by integrating objective behavioral measures, psychometric instruments, and biophysiological indicators related to stress and inhibitory control.

#### 5.2.2. Establishing a Stress Reduction and Psychological Support System for Officers

Given the high stakes, high tension, and uncertainty of police work, officers are prone to stress-induced reactions such as anxiety, depression, and emotional exhaustion. To address this issue, a multi-level institutional support strategy is proposed as follows:

1. Institutional Care Mechanism

Drawing from pandemic response models, agencies could establish a “Mental Health Service Task Force” coordinated by senior administrative leaders. This task force would oversee mental health promotion for officers, ensure cross-departmental coordination, and implement timely response and support services.

2. Tiered Psychological Support Services

We recommend establishing various intervention programs, including preliminary physical and mental health screenings, supervisor interviews, psychological referrals, thematic seminars, and individual or group psychological support services. These services should include differentiated care based on officers’ risk levels (e.g., confirmed cases, close contacts, and those at high risk of stress).

3. Strengthening Supervisors’ Proactive Support Role

Supervisors should conduct regular visits to frontline units, express genuine concern for officers’ emotional and physical well-being, listen to their challenges, and ensure the availability of support resources. Such actions contribute to a sense of organizational care and psychological safety, thereby boosting morale and trust.

4. Improving Leave Policies and Stress-Relief Facilities

**Figure 4:**
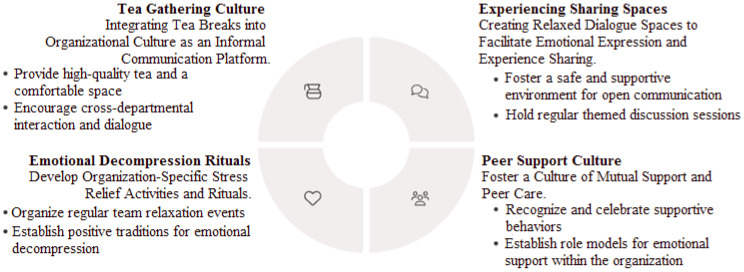
Culturally embedded informal support mechanisms.

Recruitment and promotion systems should consider candidates’ psychological traits and stress adaptability. After employment, flexible leave policies and diverse recreational facilities (e.g., gym access, leisure spaces) should be provided to help officers decompress. For those showing signs of emotional distress, additional flexible or supplementary leave options should be offered to support their mental and physical recovery. As illustrated in Figure 4, culturally embedded informal support mechanisms play a complementary role in stress relief and recovery among police officers.

#### 5.2.3. Future Work

Future research can further extend the present findings by combining qualitative insights with quantitative biophysiological indicators. The present study’s findings regarding stress, emotional regulation, and their effects on impulsive behavior among police officers could be significantly enhanced by incorporating real-time, ecologically valid measurement approaches. Recent advances in ecological momentary assessment (EMA) methodology offer promising avenues for future investigation. For instance, Bromet et al. (2020) [58] successfully employed EMA to track positive and negative affect in the daily lives of World Trade Center first responders with PTSD, demonstrating that real-time, repeated assessments can capture dynamic fluctuations in emotional states that retrospective self-reports often miss. This approach revealed that positive emotions were not uniformly inhibited across daily living even among responders with chronic PTSD, highlighting the importance of examining within-person variability in emotional experiences. Such findings underscore the value of moving beyond cross-sectional designs and retrospective recall to capture the temporal dynamics of stress, emotion regulation, and behavioral responses in police officers’ natural work environments. First, continuous or repeated measurements of heart rate variability (HRV) and heart rate—potentially integrated with EMA protocols—could be used to construct stress indices associated with different duty types, organizational contexts, and stages of police work. Combining physiological markers with real-time self-reports would enable researchers to identify moment-to-moment associations between autonomic arousal, subjective stress experiences, and emotion regulation attempts, thereby illuminating the temporal sequencing of stress-response processes. Future studies could also incorporate event-contingent EMA recordings to capture officers’ cognitive and emotional states immediately before, during, and after critical incidents, providing fine-grained data on how acute stressors impact decision-making in real-world policing contexts. Building on the approach demonstrated by Bromet et al. (2020) [58] with first responders, future research could deploy smartphone-based EMA protocols to assess police officers’ stress, emotional regulation strategy use, and behavioral outcomes multiple times daily over extended periods. Such designs would yield rich longitudinal data capable of capturing cumulative stress effects, identifying critical windows for intervention, and testing whether emotion regulation training—as suggested in our first major finding—produces measurable improvements in officers’ real-time coping capacity and well-being. In parallel, future investigations into the use of EEG, imaging, and functional imaging may further elucidate the neural mechanisms underlying stress-related decision-making and aggression-related behaviors. Finally, mixed-methods designs that integrate EMA, physiological monitoring, and qualitative interview narratives would make it possible to quantify autonomic nervous system activity, link it to officers’ subjective experiences of stress and coping, and empirically evaluate the effectiveness of the screening frameworks and intervention strategies proposed in this study.

## 6. Conclusions

In summary, this study provides a preliminary qualitative examination of frontline police officers’ experiences of occupational stress, emotional regulation, and coping processes, highlighting how individual traits, organizational stressors, and available support mechanisms intersect in everyday policing practice. By grounding the analysis in officers’ own narratives, the findings contribute context-specific insights into the challenges of stress coping and emotional regulation within contemporary police organizations and underscore the importance of organizational awareness and supportive environments.

These policy implications should be interpreted with caution and are intended to offer exploratory, context-specific insights rather than prescriptive recommendations, particularly given the qualitative design and limited sample size of the present study. Accordingly, they are best viewed as complementary to existing research, providing grounded perspectives from frontline officers rather than definitive guidance for policy implementation.

## Figures and Tables

**Table 1:** Participant Characteristics.

Participant ID	Gender	Years of Service	Rank/Position
P01	Male	15	Sergeant
P02	Female	8	Officer
P03	Male	22	Inspector
P04	Male	12	Officer
P05	Female	6	Officer
P06	Male	18	Sergeant
P07	Male	10	Officer

Note: All participants were frontline police officers. Pseudonyms or identification codes are used to protect participant confidentiality.

**Table 2:** Thematic categories of stress faced on duty: stress faced on duty.

Interviewee ID	Summary from a Stress Perspective	Verbatim Quote
01	Stress exhibits a clear pattern of “hierarchical transmission,” flowing downward from senior officials and elected representatives, with frontline officers bearing the ultimate burden.	“If the station chief is under pressure, then we’re all under pressure. Once the precinct head feels pressure from a legislator, the command passes down layer-by-layer until the frontline officer takes it all.”
02	Participant feels that stress is concentrated at the “supervisory level,” acting as both the origin and turning point of pressure within the unit.	“Under the current circumstances, all the pressure is on the supervisors.”
03	Participant identifies “self-expectations and level of personal involvement” as key sources of stress—detachment reduces perceived stress.	“If you’re committed to serving the public wholeheartedly, you’ll feel the pressure. But if you’re indifferent, then honestly, you won’t feel any pressure at all.”
04	Participant considers “self-identity and role perception” as central to how stress is experienced.	“I think the pressure depends on what type of police officer you want to be.”
05	Participant cites “excessive workload and multitasking” as major stressors in daily duties.	“There are always so many incident reports, official documents, and assignments to handle. Sometimes I even prepare presentations—I’m just overwhelmed and physically exhausted.” (Simulated supplement)
06	Participant indicates a “lack of flexible shifts and family support” as contributors to accumulating stress, particularly under a rotating shift system (Inferred case expansion).	“Sometimes I work the graveyard shift and still have to rush to court or write reports after. My family doesn’t really understand, and over time, I start to wonder if I can keep going.”
07	Participant emphasizes pressure peaks during “major emergencies,” and the aftermath, including media scrutiny, creates a sustained psychological burden (Derived from organizational stress hierarchy).	“When a major case occurs, the entire unit is on edge. Then, the media calls to ask for updates. Often the real stress doesn’t come from solving the case, but from facing the public eye.”

**Table 3:** Thematic categories of stress faced on duty: role of CCTV in police investigations and the pressure on police officers.

Thematic Category	Subcategory	Interview Data Reference	Thematic Summary
1. Heavy Dependence on CCTV	Case reconstruction and suspect identification rely on video footage.	Police officer 01; 02	CCTV serves as the core of the evidentiary chain and is one of the most critical tools in the early stages of an investigation.
2. Inadequate System Coverage	CCTV blind spots or a lack of footage lead to investigative dead ends.	Police officer 03	When footage is unavailable and other forms of evidence are absent, investigations often stall, increasing the risk of misjudgment and aggravating officers’ psychological burden.
3. Delay in Reporting Affects Access	Late reporting results in footage overwritten automatically.	Police officer 04; 05	Victims’ reporting delays frequently lead to the loss of critical CCTV data, disrupting evidentiary chains and intensifying officers’ investigative and psychological pressure.
4. Time-Consuming Retrieval Process	Complex procedures and fragmented footage across zones.		Multi-location cases require officers to cross-reference footage across numerous systems, considerably increasing both workload and time pressure.
5. Lack of Public Awareness	Victims underestimate the time sensitivity of video data.	Police officer 07	Citizens’ lack of awareness regarding storage retention periods results in the inadvertent loss of crucial evidence, indirectly heightening officers’ operational and psychological strain.

**Table 4:** Thematic categories of stress faced on duty: stress control and emotional dysregulation behaviors.

Thematic Category	Subcategory	Interview Insights	Thematic Summary
1. Internalization and Personality Traits	Reserved personality, self-suppression, poor communication.	Police officer 02; 03	Officers who struggle with expression or adjustment tend to accumulate stress internally, which may result in behavioral outbursts when triggered.
2. Externalized Conflict Behaviors	Throwing objects, issuing tickets aggressively, and direct confrontation with citizens.	Police officer 01; 02; 03	Officers with low emotional regulation tend to release stress and anger through verbal or physical aggression.
3. Retaliatory Workplace Behaviors	Sabotaging superiors, setting up situations to cause blame.	Police officer 04	When emotions cannot be voiced upward, they may manifest as passive-aggressive or retaliatory behavior, reflecting a breakdown in communication and trust within the organization.
4. Anonymous Online Venting	Posts in anonymous forums, such as anti-police pages.	Police officer 06; 07	Officers reported turning to anonymous platforms to express dissatisfaction when internal communication channels are ineffective.
5. Emotionally Repressive Organizational Culture	Pressure to “silently endure” and a lack of formal complaint mechanisms.	Police officer 06	Many respondents noted that the organizational culture discourages emotional expression and lacks sufficient psychological support or communication feedback systems.

**Table 5:** Thematic categories of stress faced on duty: coping strategies police officers employ.

Thematic Category	Subcategory	Interview Insights	Thematic Summary
1. Social Support	1.1 Peer reassurance and guidance 1.2 Emotional support from family and friends 1.3 Duty adjustment by supervisors	Police officer 01; 02; 03	Peer support provides a crucial outlet for emotion; family and friends provide everyday emotional sharing; supervisory adjustment of duties is an effective stress relief strategy.
2. Professional Support	2.1 Distrust in internal counseling and fear of stigmatization 2.2 Need for contextualized psychological training	Police officer 01; 04; 05; 06	Although most officers acknowledged the need for psychological support systems, concerns about stigma, career impact, and the relevance of training content to real-life police duties were prevalent.
3. Individual Coping	3.1 Emotional intelligence and self-guided regulation 3.2 Relaxation methods such as exercise or tea breaks 3.3 Practical skills development and mindset shift 3.4 Experience-sharing and cultural adaptation	Police officer 03; 05; 07	Emotional intelligence and self-regulation are viewed as key coping mechanisms. Cultural practices such as tea gatherings and mentorship from experienced officers help younger officers adapt and grow.

## Data Availability

The data presented in this study are available upon request from the corresponding author. The data are not publicly available due to privacy and ethical considerations.

## Abbreviations

ELS	Early life stress
PFC	Prefrontal cortex
OFC	orbitofrontal cortex
CCTV	closed-circuit television
EQ	emotional quotient
